# Genome sequences of bacteria isolated from *Pinus radiata*-associated soils

**DOI:** 10.1128/mra.01498-25

**Published:** 2026-04-20

**Authors:** Charlotte Armstrong, Mariann Eagle, Steve A. Wakelin

**Affiliations:** 1 Bioeconomy Science Institute 838278, Lincoln, New Zealand; California State University San Marcos 14673, San Marcos, California, USA

**Keywords:** soil microbiology, genomics, soil bacteria, forest soils

## Abstract

Complete bacterial genomes and functional annotations of 20 isolates from planted *Pinus radiata* forest soils in New Zealand are reported. These genomes expand the taxonomic and functional representation of forest soil microbes in genome databases, supporting future microbial ecology studies.

## ANNOUNCEMENT

Forest soils are critical to tree health and productivity, with microbes driving essential ecosystem services, such as nutrient cycling, carbon storage, and supporting biodiversity (1). Despite this importance, forest soil microbes remain largely unexplored and underrepresented in genomic databases (2), with few cultured isolates having publicly available genome sequences (3). Expanding the genomic representation of forest soil microorganisms improves taxonomic resolution and enables more accurate functional predictions (4). Here, we present annotated genomes of 20 bacterial isolates from soils planted with the conifer *P. radiata* D. Don (Monterey pine), contributing to taxonomic and functional characterization of forest soil microbiomes. Given that *Pinus radiata* is one of the most widely planted pine species globally (5), these findings have wide relevance.

Topsoil (0–10 cm depth) was collected approximately 1 m from *P. radiata* trees at multiple locations across the South Island of New Zealand between 2022 and 2024. Pure bacterial cultures were obtained by preparing a 1:100 g mL^−1^ soil-to-sterile-water slurry, shaking for 1–4 h, and serially diluting to 10^–6^−10^−8^ g soil mL^−1^. Aliquots of 100 µL were spread onto Reasoner’s 2A agar (R2A) and incubated at room temperature in the dark until single colonies formed. Most colonies appeared within 1–4 weeks; however, some slow-growing isolates required incubation for up to 31 weeks. Details of sampling location, date, GPS coordinates, forest type, soil classification (6), incubation duration, and colony characteristics are provided in Table 1.

**TABLE 1:** Summary sampling, sequencing, genome assembly, and annotation data for the eleven *Pinus radiata* associated soil bacteria

	*Pseudono-* *cardia* sp. Cha107L01	*Rhodano-* *bacter* sp. Col0626	*Myco-* *bacterium* sp. Dal123C01	*Mycolici-* *bacterium* sp. Dal123E01	*Arthro-* *bacter* sp. Hor0625	*Xylani-* *monas* sp. McL0601	*Kitasato-* *spora* sp. McL0602	*Mucilagini-* *bacter* sp. McL0603	*Aquiha-* *bitans* sp. McL0605	*Leifsonia* sp. McL0607	*Bradyrhi-* *zobium* sp. McL0615	*Bradyrhi-* *zobium* sp. McL0616	*Cellulo-* *monas* sp. McL0617	*Leifsonia*^a^ sp. McL0618	*Angusti-* *bacter* sp. McL 0619	*Dactylospo-* *rangium* sp. McL0621	*Pseudo-* *monas* sp. McL0111	*Baekduia* sp. Peel2402	*Sphingo-* *monas* sp. Tas61C01	*Dyella* sp. Tek66A03
Sampling and isolation																				
BioSample accession	SAMN5068- 8279	SAMN4786- 5726	SAMN5068- 8756	SAMN5068- 8897	SAMN5067- 3836	SAMN4777- 2641	SAMN4777- 2641	SAMN4786- 2464	SAMN4786- 3567	SAMN4786- 3626	SAMN4786- 3641	SAMN4786- 3706	SAMN4786- 5601	SAMN4786- 5664	SAMN4786- 5609	SAMN4786- 5710	SAMN5067- 3004	SAMN5074- 0841	SAMN5074- 0844	SAMN5074- 0849
Sample location	Chaneys Forest	Lake Coleridge	Dalethorpe Forest	Dalethorpe Forest	Horoata	McLeans Island	Mc- Leans Island	McLeans Island	Mc- Leans Island	Mc- Leans Island	Mc- Leans Island	McLeans Island	Mc- Leans Island	McLeans Island	Mc- Leans Island	McLeans Island	McLeans Island	Rangitata	Tasman	Tekapo
Date sampled	15/05/2024	26/04/2022	20/05/2024	20/05/2024	23/03/2022	6/05/2022	6/05/2022	6/05/2022	6/05/2022	6/05/2022	6/05/2022	6/05/2022	6/05/2022	6/05/2022	6/05/2022	6/05/2022	6/05/2022	12/09/2022	2/03/2023	1/04/2023
GPS	43.43275928 S 172.66351382 E	43.36668793 S171.53020 368 E	43.38844453 S 171.82902 571 E	43.38844453 S 171.82902 571 E	43.54437403 S171.90185 284 E	43.4636324 S172.3987 245 E	43.4636324 S172.3987 245 E	43.4636324 S 172.3987 245 E	43.4636324 S172.3987 245 E	43.4636324 S172.3987 245 E	43.4636324 S172.3987 245 E	43.4636324 S172.3987 245 E	43.4636324 S172.3987 245 E	43.4636324 S172.3987 245 E	43.4636324 S172.3987 245 E	43.4636324 S 172.3987 245 E	43.4636324 S172.3987 245 E	43.7995800 S171.2350 500 E	41.5744214 S172.7386 342 E	44.0367580 S170.4269 300 E
Forest type^b^	Commercial plantation forest	Arboretum	Commercial plantation forest	Commercial plantation forest	Mixed woodland	Commercial plantation forest	Commercial plantation forest	Commercial plantation forest	Commercial plantation forest	Commercial plantation forest	Commercial plantation forest	Commercial plantation forest	Commercial plantation forest	Commercial plantation forest	Commercial plantation forest	Commercial plantation forest	Commercial plantation forest	Commercial plantation forest	Commercial plantation forest	Commercial plantation forest
Soil classification	Typic Sandy Brown soils (BST)	Acidic Orthic Brown soils (BOA)	Typic Rocky Recent soils (RXT)	Typic Rocky Recent soils (RXT)	Typic Argillic Pallic soils (PJT)	Typic Fluvial Recent soils (RFT)	Typic Fluvial Recent soils (RFT)	Fluvial Recent soils (RFT)	Typic Fluvial Recent soils (RFT)	Typic Fluvial Recent soils (RFT)	Typic Fluvial Recent soils (RFT)	Typic Fluvial Recent soils (RFT)	Typic Fluvial Recent soils (RFT)	Typic Fluvial Recent soils (RFT)	Typic Fluvial Recent soils (RFT)	Typic Fluvial Recent soils (RFT)	Typic Fluvial Recent soils (RFT)	Acidic Firm Brown soils (BFA)	Acidic-weathered Firm Brown soils (BFWA)	Typic Orthic Brown soils (BOT)
Weeks growth until first colony appeared	20	2	6	3	2	1	1	2	3	1	3	2	3	3	3	3	19	3	31	23
Colony color	White	Yellow	Clear	Creamy	White, translucent	Creamy	Creamy	Yellow	White	Creamy	White	Creamy	Yellow/orange	White, translucent	Creamy	Creamy	Clear	White	Orange	Yellow
Sequencing																				
SRA accession Illumina	SRX30683444	SRX28489344	SRX30683446	SRX30683448	SRX30683442	SRX28299724	SRX29315281	SRX28362013	SRX28489336	SRX28489346	SRX28489348	SRX28489350	SRX28489352	SRX28489338	SRX28489340	SRX28489342	SRX30683434	SRX30683436	SRX30683438	SRX30683440
SRA accession Nanopore	SRX30683445	SRX28489345	SRX30683447	SRX30683449	SRX30683443	SRX28299725	SRX29315282	SRX28362014	SRX28489337	SRX29213834	SRX28489349	SRX28489351	SRX28489353	SRX28489339	SRX28489341	SRX29213835	SRX30683435	SRX30683437	SRX30683439	SRX30683441
No. of Illumina paired end reads	3,832,330	2,353,270	4,604,763	4,049,446	4,204,784	2,267,628	2,283,787	2,356,782	2,497,236	2,162,220	2,178,535	2,052,190	2,252,441	2,290,096	2,244,391	2,625,031	3,922,020	4,222,578	3,657,745	4,084,978
No. of Nanopore reads	527,517	112,837	296,819	479,259	698,664	265,681	281,688	109,105	147,217	175,626	150,256	107,942	161,924	148,670	83,759	185,425	1,279,479	1,258,973	377,053	304,559
Nanopore *N*_50_ (bp)	5,729	8,384	7,865	6,511	5,076	5,925	5,946	8,764	7,842	6,965	7,878	9,357	7,666	7,491	7,963	7,578	2,953	4,383	6,705	6,887
Mean nanopore read length (bp)	4,835	5,991	6,562	5,265	3,798	4,386	4,103	5,984	5,995	5,193	5,773	6,538	5,369	5,119	5,070	4,744	1,801	2,539	5,242	5,687
Max Nanopore read length (bp)	317,845	47,019	201,808	228,320	131,762	727,774	308,367	108,803	203,607	284,279	152,975	141,922	181,598	207,030	169,338	124,178	399,088	368,345	88,497	120,350
Assembly																				
No. of contigs	1	1	1	1	1	1	2 ^c^	1	1	1	1	1	1	1	1	1	1	1	1	1
Assembled genome size (bp)	9,858,910	4,322,808	5,890,649	6,083,269	3,451,454	3,919,473	7,806,759	5,653,601	4,824,042	3,892,563	8,847,784	8,636,646	4,142,206	4,224,326	4,762,090	12,704,286	6,405,117	5,518,467	3,808,993	5,009,275
Assembled genome *N*_50_ (bp)	9,858,910	4,322,808	5,890,649	6,083,269	3,451,454	3,919,473	7,790,529	5,653,601	4,824,042	3,892,563	8,847,784	8,636,646	4,142,206	4,224,326	4,762,090	12,704,286	6,405,117	5,518,467	3,808,993	5,009,275
Assembled genome GC content (%)	70.41	63.72	65.42	66.08	68.91	72.52	71.74	40.41	71.72	68.75	62.15	62.98	71.83	66.61	71.06	72.29	58.86	71.4	67.41	62.82
Assembled genome coverage (×)	373	318	567	611	1,127	469	235	239	337	399	171	152	371	341	229	131	539	805	802	586
GenBank accession	CP199579	CP188260	CP199580	CP199581	CP199586	CP187527	CP1972476, CP1972467	CP188251	CP188252	CP188253	CP188254	CP188255	CP188256	CP188258	CP188257	CP188259	CP199585	CP199582	CP199583	CP199584
Annotations																				
No. of protein coding genes	9,035	3,741	5,322	5,742	3,077	3,534	6,867	5,095	4,411	3,619	8,264	8,053	3,743	3,996	4,390	11,675	5,586	5,293	3,461	4,328
No. of tRNA, rRNA	48, 9	49, 6	47, 3	47, 6	51, 15	50, 9	72, 30	47, 6	45, 3	45, 3	74, 3	48, 3	46, 6	45, 3	46, 3	101, 6	74, 19	47, 3	49, 6	50, 6
Predicted secondary metabolite clusters	Alkaloid biosynthesis from L-lysine, auxin biosynthesis and clavulanic acid biosynthesis	Auxin biosynthesis	Alkaloid biosynthesis from L-lysine and auxin biosynthesis	Alkaloid biosynthesis from L-lysine and lanthionine synthetases	Auxin biosynthesis and alkane synthesis	None	Auxin biosynthesis and thiazole-oxazole-modified microcin (TOMM) synthesis	Alkaloid biosynthesis from L-lysine and auxin biosynthesis	Auxin biosynthesis and clavulanic acid biosynthesis	None	Alkaloid biosynthesis from L-lysine, auxin biosynthesis and thiazole-oxazole-modified microcin (TOMM) synthesis	Alkaloid biosynthesis from L-lysine, auxin biosynthesis and thiazole- oxazole-modified microcin (TOMM) synthesis	Alkane synthesis	Alkane synthesis and auxin biosynthesis	None	Lanthionine synthetases	Auxin biosynthesis	Auxin biosynthesis	Alkaloid biosynthesis from L-lysine and auxin biosynthesis	Auxin biosynthesis and clavulanic acid biosynthesis
Taxonomical placement																				
Top BLASTn match from 16S rRNA database	*Pseudonocardia xinjiangensis* strain XJ 45	*Rhodano-* *bacter* *terrae* strain GP18-1	*Mycobac-* *terium* *montefiorense* strain ATCC BAA-256	*Mycolicibac-terium* *helvum* strain JCM 30396	*Arthrobacter humicola* strain KV-653	*Xylanimonas allomyrinae* strain 2JSPR-7	*Kitasatospora mediocidica* strain NBRC 14789	*Mucilaginibacter yixingensis* strain YX-36	*Aquihabitans daechungensis* strain CH22-21	*Leifsonia soli* strain TG-S248	*Bradyrhizobium uaiense* strain UFLA03-164	*Bradyrhizobium guangdongense* strain CCBAU	*Cellulomonas chitinilytica* JCM 16,927	*Leifsonia tongyongensis* strain KIS66-7	*Angustibacter luteus* strain TT07R-79	*Dactylosporangium salmoneum* strain NRRL B-16294	*Pseudomonas germanica* strain FIT28	*Baekduia* *soli* strain BR7-21	*Sphingomonas cynarae* strain SPC-1	*Dyella halodurans* strain DHOG02
Accession number from top BLASTn match in 16S rRNA database	NR_044562.1	NR_044128.1	NR_028808.1	NR_152647.1	NR_041546.1	NR_180425.1	NR_112444.1	NR_148861.1	NR_132289.1	NR_116501.1	NR_169392.1	NR_145893.1	NR_041511.1	NR_134019.1	NR_112956.1	NR_116878.1	NR_181838.1	NR_178337.1	NR_109167.1	NR_171455.1
ANIb (%)	72.87	82.00	85.43	83.99	81.86	82.80	79.00	69.71	76.56	83.98	**79.00**	85.63	77.90	83.00	81.68	83.00	86.25	80.08	75.52	86.97

^a^ Previously classified within the *Diaminobutyricibacter* genus before being reclassified as *Leifsonia* (7).

^b^ Commercial plantation forests were exclusively planted with *P. radiata.*

^c^ The assembly consists of two circular replicons: a 7,790,529 bp chromosome and a 16,230 bp plasmid.

Genomic DNA was extracted from 50 to 100 mg cell mass using the ZymoBIOMICS DNA Miniprep Kit following the manufacturer’s recommendations. Sequencing was conducted at SeqCentre (Pittsburgh, USA) using Illumina and Oxford Nanopore platforms.

Illumina libraries were prepared using the tagmentation- and PCR-based Illumina DNA Prep Kit with IDT 10 bp unique dual indices (target insert size: 320 bp; no size selection). Sequencing on a NovaSeq 6000 generated 2 × 151 bp paired-end reads. Demultiplexing, quality control, and adapter trimming were conducted within Bcl-convert v4.1.5 (8). Default parameters were used on all software, unless otherwise specified.

Nanopore libraries were prepared using the Ligation Sequencing Kit (SQK-NBD114.24) with the NEBNext Companion Module (E7180L); no shearing or size selection was performed. Libraries were sequenced on GridION R10.4.1 flow cells in 400 bp mode (min read length: 200 bp). Basecalling and demultiplexing used Guppy v6.4.6 (9) in super-accurate mode, and adapter trimming used Porechop v0.2.3_seqan2.1.1 (10). Read statistics are presented in Table 1.

Assemblies were generated from Nanopore reads using Flye v2.9.1 (11) with the nano-hq model and polished with Illumina reads using Pilon v1.24 (12). Contigs with <15× coverage were excluded. Circularization was assessed with Circulator v1.5.5 (13), and assembly quality with QUAST v5.0.2 (14). Annotation was performed using the National Center for Biotechnology Information (NCBI) Prokaryotic Genome Annotation Pipeline v6.10 (15). Secondary metabolite gene clusters were predicted with RAST (16). Full assembly and annotation metrics are provided in Table 1.

Taxonomic assignment to genus level was determined by phylogenetic placement of the full-length 16S rRNA gene obtained by Sanger sequencing (Macrogen, South Korea), against the top 10 BLASTn v2.16.0+ hits from the NCBI RefSeq 16S rRNA database (release 229). Taxonomic placement was further confirmed using average nucleotide identity (ANI). ANIb values were calculated with JSpeciesWS v5.0.2 (17) using the closest matching genomes from the RefSeq database (Table 1).

## Data Availability

The complete and annotated genomes reported here have been deposited in GeneBank under the accession numbers provided in Table 1. The Illumina and Nanopore raw reads can be accessed under the Sequence Read Archive (SRA) accession numbers provided in Table 1. Annotations generated by RAST are available on Figshare (https://doi.org/10.6084/m9.figshare.31992378).
